# Dietary fibre intake and the risk of diverticular disease: a systematic review and meta-analysis of prospective studies

**DOI:** 10.1007/s00394-019-01967-w

**Published:** 2019-04-29

**Authors:** Dagfinn Aune, Abhijit Sen, Teresa Norat, Elio Riboli

**Affiliations:** 1 Department of Epidemiology and Biostatistics, School of Public Health, Imperial College London, St. Mary’s Campus, Norfolk Place, Paddington, London, W2 1PG UK; 2 Department of Nutrition, Bjørknes University College, Oslo, Norway; 3 Department of Endocrinology, Morbid Obesity and Preventive Medicine, Oslo University Hospital, Oslo, Norway; 4 Department of Public Health and Nursing, Faculty of Medicine and Health Sciences, Norwegian University of Science and Technology, Trondheim, Norway

**Keywords:** Fibre, Diverticular disease, Systematic review, Meta-analysis

## Abstract

**Background:**

A high intake of dietary fibre has been associated with a reduced risk of diverticular disease in several studies; however, the dose–response relationship between fibre intake and diverticular disease risk has varied, and the available studies have not been summarised in a meta-analysis. We conducted a systematic review and meta-analysis of prospective cohort studies to clarify the association between dietary fibre intake, fibre subtypes, and the risk of diverticular disease.

**Methods:**

PubMed and Embase databases were searched up to August 9th 2018. Summary relative risks (RRs) and 95% confidence intervals (CIs) were calculated using a random-effects model and nonlinear associations were modelled using fractional polynomial models.

**Results:**

Five prospective cohort studies with 19,282 cases and 865,829 participants were included in the analysis of dietary fibre and diverticular disease risk. The summary RR was 0.74 (95% CI 0.71–0.78, *I*^2^ = 0%) per 10 g/day. There was no evidence of a nonlinear association between dietary fibre intake and diverticular disease risk, *p*_nonlinearity_ = 0.35, and there was a 23%, 41% and 58% reduction in risk for an intake of 20, 30, and 40 g/day, respectively, compared to 7.5 g/day. There was no evidence of publication bias with Egger’s test, *p* = 0.58 and the association persisted in subgroup and sensitivity analyses. The summary RR per 10 g/day was 0.74 (95% CI 0.67–0.81, *I*^2^ = 60%, *n* = 4) for cereal fibre, 0.56 (95% CI 0.37–0.84, *I*^2^ = 73%, *n* = 2) for fruit fibre, and 0.80 (95% CI 0.45–1.44, *I*^2^ = 87%, *n* = 2) for vegetable fibre.

**Conclusions:**

These results suggest that a high fibre intake may reduce the risk of diverticular disease and individuals consuming 30 g of fibre per day have a 41% reduction in risk compared to persons with a low fibre intake. Further studies are needed on fibre types and risk of diverticular disease and diverticulitis.

**Electronic supplementary material:**

The online version of this article (10.1007/s00394-019-01967-w) contains supplementary material, which is available to authorized users.

## Introduction

Diverticular disease has been considered a “disease of the western civilisation” [1] due to the fact that the incidence and prevalence of diverticular disease range more than 20–40-fold between high- and low-risk populations, and tend to be more common in high-income countries, where Westernised lifestyles prevail, than in low-income countries [2, 3]. Secular trend studies have found rapid increases in the incidence of diverticular disease within countries, with rates increasing two- to fourfold between 1974 and 1986 in Japan [4]. An autopsy study reported a prevalence of 1% among Japanese in Japan, but a prevalence of 50% among Japanese in the US [5]. In addition, other migration studies have also suggested an increased risk with a longer duration since settlement [6]. In the US, 65% of adults will develop diverticulosis by age 80 years [7, 8]. Collectively, these observations suggest that modifiable risk factors are of major importance for the development of diverticular disease. Overweight and obesity [9], low physical activity [9], smoking [10] and NSAID use [11] are established risk factors for diverticular disease.

A diet low in fibre and high in red meat has been associated with increased risk of diverticular disease [12] as well as other diseases of the colon including colorectal adenomas [13], colorectal cancer [14] and Crohn’s disease [15]. Several prospective studies have consistently reported a lower risk of diverticular disease with a high fibre intake [12, 16–18]; however, the shape of the dose–response relationship has differed somewhat with some studies reporting a clear dose–response relationship with increasing benefit with higher fibre intake [12, 16, 17], while in two studies (one publication) there was no further benefit at very high intakes [18]. Data are also not entirely consistent with regard to the types of fibre that may be beneficial. In the Health Professionals Follow-up Study, there was an inverse association between fibre from fruit and vegetables, and risk of diverticular disease, but no association was observed for cereal fibre [12]. In the Million Women’s Study, there was an inverse association for fibre from fruits, vegetables, and cereals, but a positive association was observed with intake of fibre from potatoes; however, after mutual adjustment between fibre types, the inverse association with vegetable fibre disappeared [17]. In the Swedish Mammography Cohort and the Cohort of Swedish Men, fibre from fruit and vegetables were inversely associated with diverticular disease risk, but no significant association was observed with fibre from cereals [18]. To provide a better estimate of the strength and shape of the dose–response relationship between intakes of fibre and subtypes of fibre and diverticular disease risk, we therefore conducted a systematic review and meta-analysis of published prospective studies on fibre intake and risk of diverticular disease.

## Methods

### Search strategy and inclusion criteria

We (DA, AS) searched the PubMed, and Embase databases from inception up to August 9th 2018 for eligible studies as part of a larger project on risk factors for diverticular disease. The search terms used are found in the Supplementary Text. We followed standard criteria (Moose) for reporting meta-analyses [19]. In addition, we searched the reference lists of the identified publications for further studies.

### Study selection

We included published prospective cohort studies and nested case–control studies within cohorts that investigated the association between dietary fibre intake and diverticular disease risk. Adjusted estimates of the relative risk (RR) had to be available with the 95% CIs in the publication. Grey literature such as abstracts and unpublished studies were not included. A list of the excluded studies and reasons for exclusions can be found in Supplementary Table 1. DA screened PubMed and AS screened Embase, and both authors screened the 39 potentially relevant studies for final inclusion.

### Data extraction

The following data were extracted from each study: the first author’s last name, publication year, country where the study was conducted, study period, sample size, number of cases and participants, exposure, quantity of intake, RRs and 95% CIs, and variables adjusted for in the analysis. Data were extracted by one author (DA) and checked for accuracy by a second author (AS).

### Statistical methods

We calculated summary RRs (95% CIs) of diverticular disease by intake of dietary fibre using the random-effects model by DerSimonian and Laird [20] which takes into account both within- and between-study variation (heterogeneity). The average of the natural logarithm of the RRs was estimated and the RR from each study was weighted using random-effects weights [20]. Linear dose–response analyses were conducted using the method of Greenland and Longnecker [21] and study-specific linear trends and 95% CIs were computed from the natural log of the RRs and CIs across categories of fibre intake per day. The linear dose–response analysis was conducted on a continuous scale with an increment of 10 g/day. A potential nonlinear association was investigated using fractional polynomial models [22] and we determined the best fitting second order fractional polynomial regression model, defined as the one with the lowest deviance. A likelihood ratio test was used to test for nonlinearity [23].

Heterogeneity between studies was evaluated using *Q* and *I*^2^ statistics [24]. *I*^2^ is a measure of how much of the heterogeneity that is due to between-study variation rather than chance. *I*^2^ values of 25%, 50% and 75% indicates low, moderate and high heterogeneity, respectively. We conducted main analyses (all studies combined) and stratified by study characteristics such as duration of follow-up, sex, sample size, number of cases, geographic location, study quality and by adjustment for confounding factors to investigate potential sources of heterogeneity. Study quality was assessed using the Newcastle–Ottawa scale which rates studies according to selection, comparability and outcome assessment with a score range from 0 to 9 [25]. In sensitivity analyses, we also repeated the analyses using fixed-effects models. We calculated 95% prediction intervals (95% PIs) for the random-effects analyses. The 95% PIs further account for heterogeneity and show the range in which 95% of future studies will lie.

Publication bias was assessed using Egger’s test [26] and Begg–Mazumdar’s test [27] and by inspection of funnel plots. The statistical analyses were conducted using the software package Stata, version 13.1 software (StataCorp, Texas, US).

## Results

**Fig. 1:**
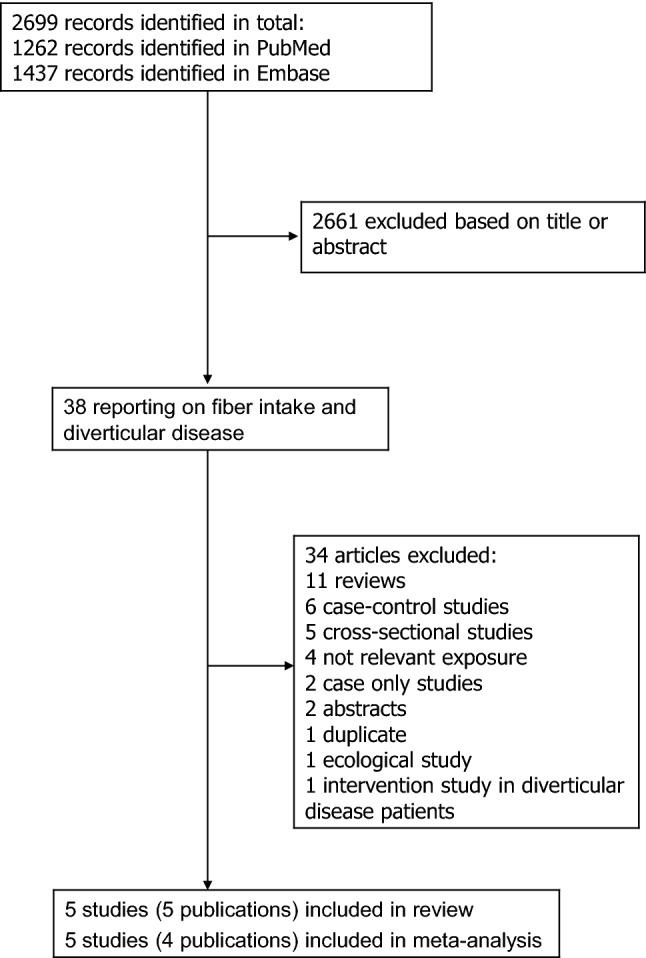
Flow-chart of study selection of dietary fibre and diverticular disease

We identified five cohort studies (four publications) [12, 16–18], that could be included in the meta-analysis of dietary fibre intake and diverticular disease (Fig. 1, Table 1). Only one study reported on dietary fibre intake and risk of diverticulitis [28] and a meta-analysis was, therefore, not possible on this outcome (Table 1). Four studies were from Europe and one study was from the USA.

**Table 1:** Prospective studies of dietary fibre and diverticular disease incidence

Author, publication year, country	Study name	Follow-up period	Study size, gender, age, number of cases	Dietary assessment	Exposure	Description of quantiles of categories	RR (95% CI)	Adjustment for confounders
Aldoori WH et al., 1994, USA	Health Professionals Follow-up Study	1986–1990, 4 years follow-up	47,888 men, age 40–75 years: 385 symptomatic diverticular disease cases	Validated FFQ, 131 food items	Total dietary fibre	13.0 g/day	1.00	Age, physical activity, energy-adjusted total fat
						18.0	0.79 (0.58–1.07)	
						21.0	0.77 (0.57–1.05)	
						24.0	0.87 (0.64–1.17)	
						32.0	0.58 (0.41–0.83)	
					Total crude fibre	3.3 g/day	1.00	
						4.5	0.81 (0.59–1.10)	
						5.4	0.76 (0.57–1.02)	
						6.4	0.78 (0.57–1.06)	
						8.3	0.54 (0.38–0.76)	
					Fruit fibre	1.1 g/day	1.00	
						2.3	1.10 (0.82–1.47)	
						3.8	0.90 (0.66–1.23)	
						5.5	0.60 (0.43–0.85)	
						8.8	0.61 (0.43–0.87)	
					Vegetable fibre	3.4 g/day	1.00	
						5.1	0.96 (0.70–1.31)	
						6.6	1.00 (0.73–1.36)	
						8.6	0.87 (0.63–1.20)	
						12.4	0.59 (0.41–0.86)	
					Cereal fibre	1.7 g/day	1.00	
						3.7	1.23 (0.90–1.67)	
						5.7	1.08 (0.78–1.49)	
						8.2	0.87 (0.61–1.22)	
						13.5	1.06 (0.76–1.47)	
Crowe FL et al., 2011, United Kingdom	EPIC-Oxford	1993–1999–2009, 11.6 years follow-up	47,033 men and women, age ≥ 20 years: 812 diverticular disease cases	Validated FFQ, 130 food items	Dietary fibre (Englyst)	<14.0/< 14.0 g/day	1.00	Age, sex, method of recruitment, region of residence, smoking, Townsend deprivation index, hyperlipidemia, receiving long term medical treatment, OC use, HRT, BMI, energy intake
						14.0 to < 17.5/14.0 to < 17.5	0.86 (0.69–1.06)	
						17.5 to < 21.2/17.5 to < 20.9	0.76 (0.61–0.96)	
						21.2 to < 26.1/20.9 to < 25.5	0.72 (0.57–0.92)	
						≥ 26.1/≥ 25.5	0.59 (0.46–0.78)	
Crowe FL et al., 2014, United Kingdom	Million Women’s Study	1996–2001–2008, 6 years follow-up	690,075 women, age 50–65 years: 17,325 diverticular disease cases	Validated FFQ, 40 food items	Dietary fibre (Englyst)	7.5 g/day	1.00 (0.96–1.04)	Age, SES, smoking, alcohol, BMI, height, HT use, total energy, type of meat consumed
						11.0	0.92 (0.89–0.95)	
						13.4	0.88 (0.85–0.91)	
						16.0	0.84 (0.81–0.87)	
						21.0	0.75 (0.72–0.78)	
						Per 5 g/day	0.86 (0.84–0.88)	
					Cereal fibre	2.7 g/day	1.00 (0.97–1.04)	+ adjusted for other fibre types
						4.5	0.96 (0.94–0.99)	
						5.7	0.91 (0.88–0.94)	
						7.1	0.86 (0.83–0.89)	
						8.9	0.80 (0.77–0.83)	
						Per 5 g/day	0.81 (0.78–0.85)	
						Per 5 g/day	0.84 (0.81–0.88)	
					Fruit fibre	1.2 g/day	1.00 (0.97–1.03)	+ adjusted for other fibre types
						2.1	0.89 (0.87–0.92)	
						2.8	0.88 (0.85–0.91)	
						3.5	0.85 (0.82–0.88)	
						5.3	0.79 (0.76–0.82)	
						Per 5 g/day	0.77 (0.73–0.82)	
						Per 5 g/day	0.81 (0.77–0.86)	
					Vegetable fibre	1.7 g/day	1.00 (0.97–1.03)	+ adjusted for other fibre types
						2.1	0.94 (0.91–0.97)	
						2.5	0.97 (0.93–1.00)	
						2.9	0.95 (0.92–0.98)	
						4.0	0.94 (0.91–0.97)	
						Per 5 g/day	0.90 (0.82–0.99)	
						Per 5 g/day	1.03 (0.93–1.14)	
					Potato fibre	1.7 g/day	1.00 (0.97–1.03)	+ adjusted for other fibre types
						2.1	1.02 (0.99–1.06)	
						2.1	1.06 (1.03–1.10)	
						2.2	1.09 (1.05–1.13)	
						2.4	1.14 (1.10–1.18)	
						Per 5 g/day	1.07 (1.05–1.10)	
						Per 5 g/day	1.04 (1.02–1.07)	
Liu PH et al., 2017, USA	Health Professionals Follow-up Study	1986–2012, 16.8 years follow-up	45,203 men, age 40–75 years: 907 diverticulitis cases	Validated FFQ 131–148 food items	Dietary fibre	15 g/day	1.00	Age, total energy, aspirin use, NSAID use, acetaminophen, red meat, vigorous physical activity, BMI, smoking
						NA	1.00 (0.82–1.21)	
						22	1.02 (0.83–1.24)	
						NA	0.88 (0.71–1.09)	
						34	0.77 (0.60–0.98)	
Mahmood MW et al., 2018, Sweden	Swedish Mammograph Cohort	1997–2005, 8.0 years follow-up	36,110 women, age 49–83 years: 505 diverticular disease cases	Validated FFQ, 96 food items	Total fibre intake	16.15 g/day	1.00	Age, smoking, BMI, education, alcohol, physical activity, hypertension, diabetes mellitus, steroid use
						19.94	0.93 (0.71–1.20)	
						23.10	0.77 (0.58–1.01)	
						28.29	0.75 (0.57–0.99)	
					Fruit and vegetable fibre	4.10 g/day	1.00	
						6.46	0.88 (0.68–1.14)	
						8.74	0.68 (0.52–0.90)	
						12.56	0.70 (0.53–0.92)	
					Cereal fibre	7.44 g/day	1.00	
						10.10	0.89 (0.67–1.17)	
						12.35	0.92 (0.70–1.22)	
						15.95	0.90 (0.68–1.19)	
Mahmood MW et al., 2018, Sweden	Cohort of Swedish Men	1997–1998–2005, 7.6 years follow-up	44,723 men, age 45–79 years: 255 diverticular disease cases	Validated FFQ, 96 food items	Total fibre intake	21.51 g/day	1.00	Age, smoking, BMI, education, alcohol, physical activity, hypertension, diabetes mellitus, steroid use
						27.54	0.76 (0.54–1.05)	
						32.38	0.79 (0.56–1.10)	
						39.63	0.61 (0.43–0.88)	
					Fruit and vegetable fibre	2.85 g/day	1.00	
						4.84	0.96 (0.69–1.34)	
						6.82	0.90 (0.64–1.26)	
						10.257	0.67 (0.46–0.98)	
					Cereal fibre	12.83 g/day	1.00	
						17.81	1.07 (0.77–1.47)	
						22.12	0.74 (0.52–1.06)	
						28.82	0.76 (0.53–1.10)	

*BMI* body mass index, *HRT* hormone replacement therapy, *HT* hormone therapy, *NA* not available, *NSAID* non-steroidal anti-inflammatory drugs, *OC* use oral contraceptive use, *SES* socio-economic status

**Fig. 2:**
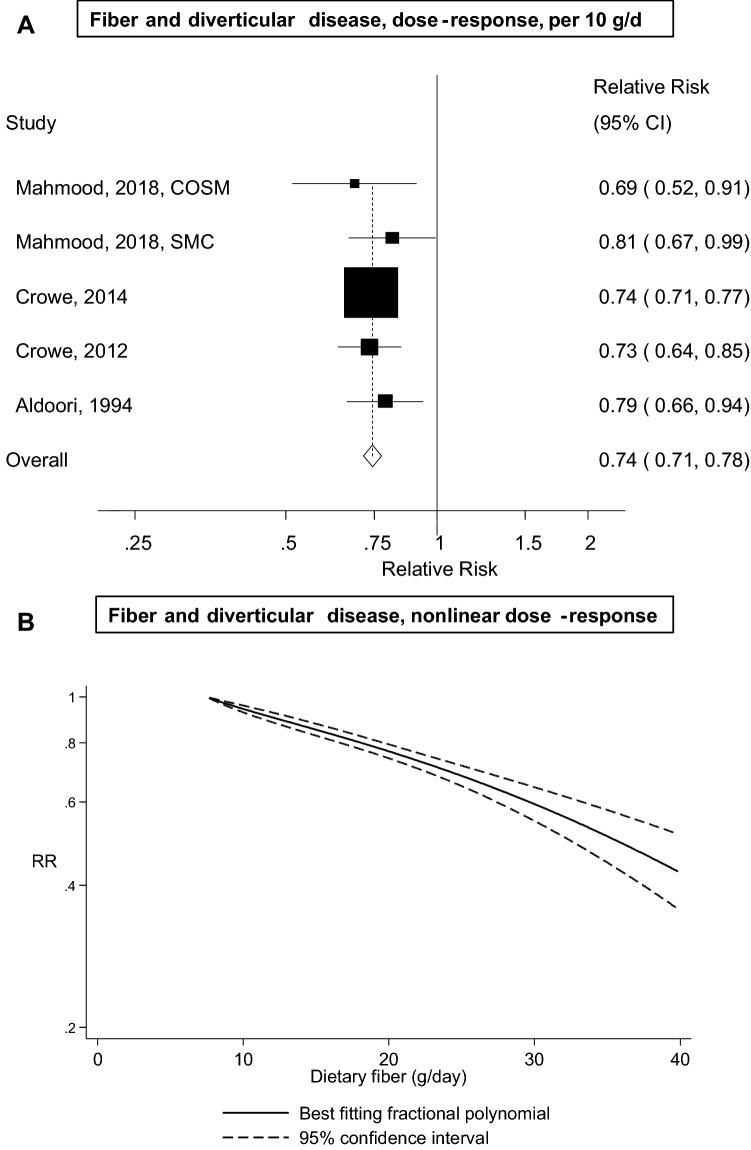
Dietary fibre and diverticular disease incidence, linear dose–response analysis (per 10 g/day) and nonlinear dose–response analysis

Five cohort studies [12, 16–18] with 19,282 cases and 865,829 participants were included in the analysis of dietary fibre intake and diverticular disease. The summary RR (95% CI) per 10 g/day was 0.74 (95% CI 0.71–0.78, *I*^2^ = 0%, *p*_heterogeneity_ = 0.80) (Fig. 2a). In sensitivity analyses excluding one study at a time, the summary RR ranged from 0.74 (95% CI 0.71–0.77) when excluding the Swedish Mammography Cohort [18] to 0.76 (95% CI 0.69–0.83) when excluding the Million Women’s Study [17] (Supplementary Fig. 1). The 95% PI also excluded the null value, summary RR 0.74 (95% PI 0.70–0.80). There was no evidence of publication bias with Egger’s test (*p* = 0.58), Begg’s test (*p* = 0.81) or by inspection of the funnel plots (Supplementary Fig. 2). There was no evidence of a nonlinear association between dietary fibre intake and diverticular disease risk (*p*_nonlinearity_ = 0.35), and the summary RRs were 0.77 (95% CI 0.74–0.79), 0.59 (95% CI 0.55–0.64) and 0.42 (95% CI 0.35–0.51) for an intake of 20, 30, and 40 g/day, respectively, compared with 7.5 g/day (Fig. 2b, Supplementary Table 2).

### Types of fibre

**Fig. 3:**
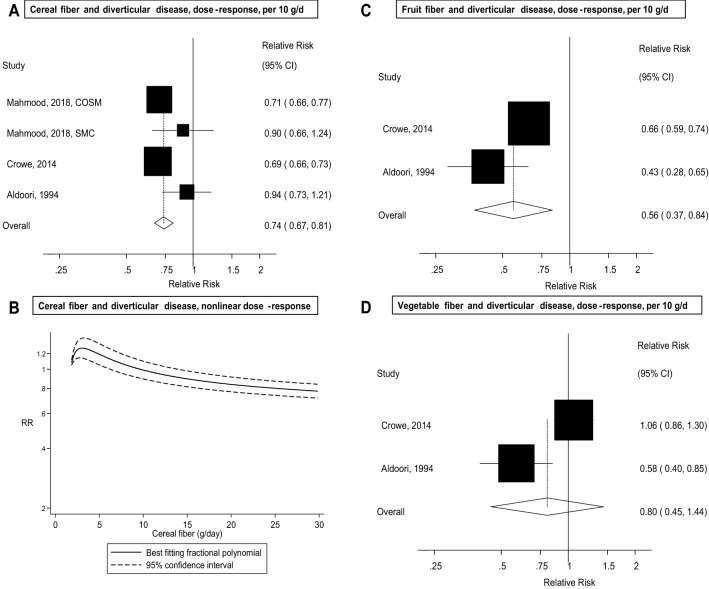
Fibre types and diverticular disease, linear and nonlinear dose–response

Four studies (three publications) [12, 17, 18] (18,470 cases, 818,796 participants), two studies [12, 17] (17,710 cases, 737,963 participants), and two studies [12, 17] (17,710 cases, 737,963 participants) were included in the meta-analysis of cereal fibre, fruit fibre, and vegetable fibre and risk of diverticular disease, respectively. The summary RR per 10 g/day of intake was 0.74 (95% CI 0.67–0.81, *I*^2^ = 60%, *p*_heterogeneity_ = 0.06) for cereal fibre intake (Fig. 3a); however, the 95% PI did not exclude the null value, summary RR 0.74 (95% PI 0.51–1.07). There was evidence of a nonlinear association between cereal fibre intake and diverticular disease risk (*p*_nonlinearity_ = 0.002), with a slight increase in risk at low levels of intake, but a reduced risk was observed from around 15 g/day up to 30 g/day (Fig. 3b). The summary RR was 0.56 (95% CI 0.37–0.84, *I*^2^ = 73%, *p*_heterogeneity_ = 0.06) per 10 g/day of fruit fibre (Fig. 3c) and 0.80 (95% CI 0.45–1.44, *I*^2^ = 87%, *p*_heterogeneity_ = 0.10) for vegetable fibre (Fig. 3d).

### Subgroup and sensitivity analyses and study quality

The inverse association between dietary fibre intake and diverticular disease persisted in a number of subgroup analyses defined by duration of follow-up, sex, geographic location, number of cases, study quality and adjustment for potential confounding factors (including age, education, alcohol, smoking, BMI, physical activity, diabetes, meat intake, energy intake) (Table 2); however, no studies adjusted for aspirin or NSAID use. With meta-regression analyses, there was no indication of heterogeneity between any of the subgroup analyses.

**Table 2:** Subgroup analyses of fibre intake and diverticular disease

	Fibre				
	*n*	RR (95% CI)	*I*^*2*^ (%)	*p*_h_^a^	*p*_h_^b^
All studies	5	0.74 (0.71–0.78)	0	0.80	
Sex					
Men	3	0.77 (0.68–0.88)	0	0.64	0.61
Women	3	0.74 (0.71–0.78)	0	0.62	
Men and women	0				
Assessment of diet					
Validated	5	0.74 (0.71–0.78)	0	0.80	NC
Not validated	0				
Duration of follow-up					
< 10 years follow-up	4	0.74 (0.71–0.78)	0	0.66	0.87
≥ 10 years follow-up	1	0.73 (0.64–0.85)			
Geographic location					
Europe	4	0.74 (0.71–0.77)	0	0.76	0.54
America	1	0.79 (0.66–0.94)			
Asia	0				
Number of cases					
Cases < 500	2	0.76 (0.65–0.88)	0	0.41	0.66
Cases 500 < 1000	2	0.76 (0.68–0.86)	0	0.41	
Cases ≥ 1000	1	0.74 (071–0.77)			
Study quality					
0–3 points	0				NC
4–6	0				
7–9	5	0.74 (0.71–0.78)	0	0.80	
Adjustment for confounders					
Age					
Yes	5	0.74 (0.71–0.78)	0	0.80	NC
No	0				
Education					
Yes	2	0.77 (0.65–0.91)	0	0.33	0.70
No	3	0.74 (0.71–0.77)	0	0.77	
Alcohol					
Yes	3	0.74 (0.71–0.78)	0	0.56	0.77
No	2	0.76 (0.68–0.85)	0	0.53	
Smoking					
Yes	4	0.74 (0.71–0.77)	0	0.76	0.54
No	1	0.79 (0.66–0.94)			
Diabetes					
Yes	2	0.77 (0.65–0.91)	0	0.33	0.70
No	3	0.74 (0.71–0.77)	0	0.77	
Aspirin use					
Yes	0				NC
No	5	0.74 (0.71–0.78)	0	0.80	
NSAID use					
Yes	0				NC
No	5	0.74 (0.71–0.78)	0	0.80	
Body mass index					
Yes	4	0.74 (0.71–0.77)	0	0.76	0.54
No	1	0.79 (0.66–0.94)			
Physical activity					
Yes	3	0.78 (0.69–0.88)	0	0.61	0.48
No	2	0.74 (0.71–0.77)	0	0.93	
Meat					
Yes	1	0.74 (0.71–0.77)			0.63
No	4	0.76 (0.69–0.83)	0	0.71	
Energy					
Yes	2	0.74 (0.71–0.77)	0	0.93	0.48
No	3	0.78 (0.69–0.88)	0	0.61	

*N* denotes the number of risk estimates

^a^*p* for heterogeneity within each subgroup

^b^*p* for heterogeneity between subgroups

We also repeated all analyses using fixed-effects models, but the results were in general similar with summary RRs of 0.74 (95% CI 0.71–0.78) for dietary fibre, 0.71 (95% CI 0.68–0.74) for cereal fibre, 0.64 (95% CI 0.57–0.71) for fruit fibre and 0.93 (95% CI 0.77–1.11) for vegetable fibre.

The mean (median) study quality scores were 8.0 (8.0) for the studies included in the analysis of dietary fibre and diverticular disease risk. With regard to the study quality scores, studies had less than maximum scores because of not being representative of the general population (two studies) and because loss to follow-up was not reported (three studies) (Supplementary Table 3).

## Discussion

This meta-analysis of five prospective studies with > 19,000 cases and > 865,000 participants suggest that a high intake of dietary fibre reduces the risk of developing diverticular disease. There was a 26%, 44%, and 26% reduction in risk per 10 g/day of intake of total dietary fibre, fruit fibre, and cereal fibre, respectively. There was no evidence of nonlinearity in the analysis of overall fibre intake and risk was reduced by 58% with an intake of 40 g/day compared with 7.5 g/day. There was some evidence of nonlinearity in the analysis of cereal fibre intake, with a slight increase in risk at low levels of intake, but a reduced risk with an intake from 15 to 30 g/day.

Limitations of the present systematic review and meta-analysis include the possibility of confounding, heterogeneity between studies, measurement errors in the assessment of dietary fibre intake and regression dilution bias during follow-up, as well as potential publication biases. Individuals with a high fibre intake tend to have a healthier overall lifestyle than individuals with a low fibre intake, with a lower intake of meat, higher physical activity, less obesity and lower prevalence of smoking. However, the inverse association between fibre intake and diverticular disease was observed across all subgroup analyses stratified by sex, duration of follow-up, geographic location, number of cases and adjustment for confounding factors (age, education, alcohol, smoking, diabetes, BMI, physical activity, meat and energy intake) and there was no between-subgroup heterogeneity. In addition, in the Million Women’s Study, the inverse association between dietary fibre intake and diverticular disease risk was observed across strata of age, socioeconomic status, smoking status, alcohol intake, BMI, height, hormone therapy use, consumption of red and processed meat, and persisted after excluding the first 3 years of follow-up (to reduce the potential for reverse causation) [17]. Nevertheless, residual confounding or confounding by other risk factors cannot be completely excluded. In the analysis of dietary fibre intake, there was no evidence of heterogeneity between studies, but there was some evidence of heterogeneity in the analyses of fruit, vegetable and cereal fibre intake. This appeared to be mainly due to differences between studies in the strength of the association between different fibre types and diverticular disease risk. This is less problematic than if there were differences in the direction of the observed effect between studies.

Measurement errors in the assessment of dietary fibre intake may have affected the observed results; however, none of the included studies made corrections for measurement error. Nevertheless, all studies used validated the food frequency questionnaires and in the Nurses’ Health Study and Health Professionals Follow-up Study, the correlations with fibre intakes estimated from food records were in the range of 0.51–0.58 [29, 30] and in the Swedish Mammography Cohort, the correlation between the FFQ and diet records was 0.4–0.7 for foods high in fibre (fruits, vegetables, whole grains) [18] and 0.71 for dietary fibre [31]. The participants may also have changed their intake of dietary fibre during follow-up; however, the studies included used a baseline dietary assessment for the analyses of dietary fibre intake and diverticular disease risk (although some of the studies may have repeated measures of dietary intake, but may not have utilised this either because of short follow-up or other reasons). However, both measurement errors and changes in diet during follow-up would most likely have led to attenuation of the relative risks given the prospective design of the studies.

A further limitation is that most of the primary studies identified diverticular disease cases by linkage to hospital databases and death registries [16–18]. One study identified symptomatic diverticular disease cases by self-report which then was validated against medical records and found 95% agreement between the two methods [32]. Most of the cases in these studies would, therefore, have been symptomatic and represent more severe disease, while it is known that many cases can be asymptomatic or only have mild symptoms. If people with a low fibre intake were more likely to be admitted to hospital and undergo examinations for symptoms like constipation, it is possible that detection bias partly could explain the observed association. However, in the Million Women’s Study, the association was similar among people with and without constipation and the association persisted also among participants without comorbidity [17]. Although publication bias can affect the results of meta-analyses of published studies, we found no evidence of such bias in this meta-analysis.

Several mechanisms may explain the inverse association between dietary fibre intake and the risk of diverticular disease. A high intake of dietary fibre is associated with increased stool bulk and reduced transit time [33–35] and may require less pressure during defecation, and may therefore reduce the possibility for the mucosa to herniate through the weak areas in the colon [1]. Bacterial fermentation of dietary fibre produces short-chain fatty acids (e.g. butyrate) which are a fuel source for the colonic cells [35]. Several animal studies have shown that a low-fibre diet substantially increases the rates for colonic diverticula [36–39]. Some studies have suggested differences in the gut microbiota of diverticular disease patients compared to controls [40–45] and one study suggested that patients with diverticular disease have depletion of microbiota with anti-inflammatory activity [40]. Further studies are needed to clarify whether and how fibre intake might interact with the microbiota in reducing diverticular disease risk. Dietary fibre is also associated with less adiposity and lower risk of overweight and obesity [46, 47] and could, therefore, reduce the risk of diverticular disease indirectly, since overweight and obesity increase the risk [9]. However, most of the studies included in this analysis adjusted for BMI; thus, it seems that the observed association is largely independent of adiposity.

The present meta-analysis has several strengths including the prospective design of the studies (which avoids recall bias and reduces the potential for selection bias), the detailed subgroup, sensitivity, and dose–response analyses, the relatively large sample size providing a robust estimate of the association between dietary fibre and diverticular disease, as well as the high study quality of the included studies. Our findings have important clinical and public health implications as the inverse association between fibre intake and diverticular disease was rather strong and dose dependent with a 41% reduction in risk at an intake of 30 g/day (compared to 7.5 g/day) and because the mean fibre intake in many populations is only around 13–25 g/day [48, 49]. The current findings are consistent with other studies showing benefits of a high fibre intake in relation to breast cancer [50], colorectal cancer [14], type 2 diabetes [51] and mortality [52], but provide further evidence of an association also with diverticular disease. Any future studies should further clarify the association between different sources of fibre and risk of diverticular disease as well as diverticulitis.

In conclusion, this meta-analysis found a strong and linear reduction in risk of diverticular disease with a high dietary fibre intake up to an intake of 40 g/day. Fibre from cereals and fruit were associated with reduced risk, but further studies are needed on fibre types given the limited number of studies. These results support public health recommendations to increase the intake of dietary fibre in the general population.

## Electronic supplementary material

Below is the link to the electronic supplementary material.

Supplementary material 1 (DOCX 38 kb)

Supplementary material 2 (DOCX 19 kb)
