# Supplementary material for: Dietary fibre intake and the risk of diverticular disease: a systematic review and meta-analysis of prospective studies
Source: Eur J Nutr. 2019 Apr 29;59(2):421–32. doi: 10.1007/s00394-019-01967-w (PMC7058673; doi:10.1007/s00394-019-01967-w)
Supplement: Supplementary file 1 — Supplementary material 1 (DOCX 38 kb) [file 394_2019_1967_MOESM1_ESM.docx]

Supplementary Text: PubMed search

((((((((“body mass index” OR BMI OR overweight OR obesity OR anthropometry OR fatness OR “body fatness” OR “abdominal fatness” OR “abdominal obesity” OR “waist circumference” OR “waist-to-hip ratio” OR “waist-to-height ratio” OR “hip circumference” OR adiposity OR weight OR "weight gain" OR "weight change" OR “weight loss” OR “body size”)) OR (“physical activity” OR exercise OR sports OR walking OR biking OR running OR fitness OR “exercise test” OR inactivity OR sedentary)) OR (Fibre OR fibre OR diet)) OR (Meat OR “red meat” OR “processed meat” OR beef OR pork OR lamb) OR (smoking OR smoke OR tobacco OR snus OR snuff OR "environmental tobacco smoke” OR “passive smoking” OR “smoking cessation")) OR (“Risk factors” OR “risk factor”))) AND (“diverticular disease” OR diverticulitis OR “diverticular bleeding” OR diverticula OR diverticulosis)) AND (“case-control” OR retrospective OR cohort OR cohorts OR prospective OR longitudinal OR “follow-up” OR “cross-sectional” OR trial OR "odds ratio" OR "relative risk" OR "hazard ratio" OR "incidence rate ratio" OR "risk ratio")

Embase search

(physical activity OR exercise OR sports OR walking OR biking OR running OR fitness OR exercise test OR inactivity OR sedentary

OR

physical activity/ OR exercise/ OR sports/ OR walking/ OR biking/ OR running/ OR fitness/ OR exercise test/ OR inactivity/ OR sedentary/

OR

body mass index OR overweight OR obesity OR anthropometry OR fatness OR body fatness OR abdominal fatness OR abdominal obesity OR waist circumference OR waist-to-hip ratio OR waist-to-height ratio OR hip circumference OR adiposity OR weight OR weight gain OR weight change OR weight loss OR body size

OR

body mass index/ OR overweight/ OR obesity/ OR anthropometry/ OR fatness/ OR body fatness/ OR abdominal fatness/ OR abdominal obesity/ OR waist circumference/ OR waist-to-hip ratio/ OR waist-to-height ratio/ OR hip circumference/ OR adiposity/ OR weight/ OR weight gain/ OR weight change/ OR weight loss/ OR body size/

OR

Meat OR red meat OR processed meat OR beef OR pork OR lamb

OR

Meat/ OR red meat/ OR processed meat/ OR beef/ OR pork/ OR lamb/

OR

Fibre OR fibreOR diet

OR

Fibre/ OR fibre/OR diet/

OR

smoking OR smoke OR tobacco OR snus OR snuff OR environmental tobacco smoke OR passive smoking OR smoking cessation

OR

smoking/ OR smoke/ OR tobacco/ OR snus/ OR snuff/ OR environmental tobacco smoke/ OR passive smoking/ OR smoking cessation/

OR

Risk factors OR risk factor

OR

Risk factors/ OR risk factor/)

AND

(diverticular disease OR diverticulitis OR diverticular bleeding OR diverticula OR diverticulosis

OR

diverticular disease/ OR diverticulitis/ OR diverticular bleeding/ OR diverticula/ OR diverticulosis/)

AND

(case-control OR retrospective OR cohort OR cohorts OR prospective OR longitudinal OR follow-up OR cross-sectional OR trial OR odds ratio OR relative risk OR hazard ratio OR incidence rate ratio OR risk ratio)

Supplementary Table 1. List of excluded studies and exclusion reasons

| Reference number | Exclusion reason |
| --- | --- |
| Abstract | (1;2) |
| Case-control study | (3-8) |
| Case only study | (9;10) |
| Cross-sectional study | (11-15) |
| Duplicate | (16) |
| Ecological study | (17) |
| Not relevant exposure | (18-21) |
| Treatment of diverticular disease patients | (22) |
| Review | (23-33) |

Reference List

1. Liu P-H, Cao Y, Strate LL et al. Adherence to a healthy lifestyle reduces risk of diverticulitis among men. Gastroenterology Conference: Digestive Disease Week 2017;5(Suppl. 1): S943.

2. Tawil J, Maynat A, Mejia MR et al. Is there any association between diverticulosis and fibre intake, demographic factors, body mass index, nonsteroidal anti-inflammatory drugs, acetylsalicylic acid and colonic cleansing? Gastrointestinal Endoscopy Conference: Digestive Diease Week, DDW 2017;85;5(Suppl.):AB266-7.

3. Manousos O, Day NE, Tzonou A et al. Diet and other factors in the aetiology of diverticulosis: an epidemiological study in Greece. Gut 1985;26:544-9.

4. Nagahashi M, Yamazaki N, Ohi G et al. [Dietary fibre intake and diverticular disease of the colon--a case control study]. Nihon Eiseigaku Zasshi 1985;40:781-8.

5. Lin OS, Soon MS, Wu SS, Chen YY, Hwang KL, Triadafilopoulos G. Dietary habits and right-sided colonic diverticulosis. Dis Colon Rectum 2000;43:1412-8.

6. Jeyarajah S, Ewence A, Chana S, Kukreja N, Papagrigoriadis S. Patients with colonoscopic findings of diverticulosis do not demonstrate decreased fibre intake or abnormal cleveland constipation score. Colorectal Disease Conference: Association of Coloproctology of Great Britain and Ireland Annual Meeting Harrogate United Kingdom Conference Start: 2009;136(5, Suppl. 1):A216.

7. Farrar WD, Deppe LM, Decker MS, Yorde KA, Kalmes K, Knapp E. Hypertension and aging are associated with colonic diverticulosis, but not fibre intake. Gastroenterology Conference: Digestive Disease Week 2017;152(5, Suppl. 1):S646-7.

8. Yang F, Zheng Y, Jiang X et al. Sex differences in risk factors of uncomplicated colonic diverticulosis in a metropolitan area from Northern China. Sci Rep 2018;8:138.

9. Leahy AL, Ellis RM, Quill DS, Peel ALG. High fibre diet in symptomatic diverticular disease of the colon. Annals of the Royal College of Surgeons of England 1985;67 (3): 173-174

10. Orhstein MH, Littlewood ER, McLean B, I. Are fibre supplements really necessary in diverticular disease of the colon? A controlled clinical trial. British Medical Journal 1981;282 (6273):1353-1356

11. Song JH, Kim YS, Ok KS, Ryu SH, Lee JH, Moon JS. Clinical characteristics of colonic diverticulosis in Korea: A prospective study. Korean Journal of Internal Medicine 2010;25 (2): 140-6.

12. Peery AF, Sandler RS, Ahnen DJ et al. Constipation and a low-fibre diet are not associated with diverticulosis. Clin Gastroenterol Hepatol 2013;11:1622-7.

13. Peery AF, Barrett PR, Park D et al. A high-fibre diet does not protect against asymptomatic diverticulosis. Gastroenterology 2012;142:266-72.

14. Peery AF, Barrett PR, Park D et al. Dietary fibre is not associated with diverticulosis. Gastroenterology Conference: Digestive Disease Week, DDW 2011;140(5, Suppl. 1): S61.

15. Afonso M, Pinto J, Veloso R, Freitas T, Carvalho J, Fraga J. Obesity and visceral fat in diverticular disease of the colon. American Journal of Gastroenterology Conference: 75th Annual Scientific Meeting of the American College of Gastroenterology San Antonio, TX United States Conference Start: 2010;105:S140.

16. Aldoori WH, Giovannucci EL, Rimm EB, Wing AL, Trichopoulos DV, Willett WC. A prospective study of diet and the risk of symptomatic diverticular disease in men. Am J Clin Nutr 1994;60:757-64.

17. Munakata A, Nakaji S, Takami H, Nakajima H, Iwane S, Tuchida S. Epidemiological evaluation of colonic diverticulosis and dietary fibre in Japan. Tohoku J Exp Med 1993;171:145-51.

18. Strate LL, Liu YL, Syngal S, Aldoori WH, Giovannucci EL. Nut, corn, and popcorn consumption and the incidence of diverticular disease. JAMA - Journal of the American Medical Association 2008;300 (8): 907-914.

19. Lim JH, Kim YS, Lee JE et al. Is development of right colonic diverticulosis associated with dietary pattern? Clinical Nutrition Conference: 38th European Society for Clinical Nutrition and Metabolism Congress, ESPEN Denmark 2016;35 (Supplement 1): S67-S68.

20. Strate LL, Keeley BR, Cao Y, Wu K, Giovannucci E, Chan AT. Major dietary patterns and risk of incident diverticulitis. Gastroenterology Conference: Digestive Disease Week 2016;April.

21. Strate LL, Keeley BR, Cao Y, Wu K, Giovannucci EL, Chan AT. Western Dietary Pattern Increases, Whereas Prudent Dietary Pattern Decreases, Risk of Incident Diverticulitis in a Prospective Cohort Study. Gastroenterology 2017;152(5):1023-1030.

22. Lahner E, Esposito G, Zullo A et al. High-fibre diet and Lactobacillus paracasei B21060 in symptomatic uncomplicated diverticular disease. World Journal of Gastroenterology 2012;18 (41): 5918-5924.

23. Talbot JM. Role of dietary fibre in diverticular disease and colon cancer. Fed Proc 1981;40:2337-42.

24. Aldoori WH. The protective role of dietary fibre in diverticular disease. Adv Exp Med Biol 1997;427:291-308.

25. Nakaji S, Danjo K, Munakata A et al. Comparison of etiology of right-sided diverticula in Japan with that of left-sided diverticula in the west. International Journal of Colorectal Disease 2002;17 (6): 365-373.

26. Trepel F. [Dietary fibre: more than a matter of dietetics. II. Preventative and therapeutic uses]. Wien Klin Wochenschr 2004;116:511-22.

27. Eglash A, Lane CH, Schneider DM. Clinical inquiries. What is the most beneficial diet for patients with diverticulosis? The Journal of family practice 2006;55 (9): 813-815.

28. Anderson JW, Baird P, Davis RH, Jr. et al. Health benefits of dietary fibre. Nutr Rev 2009;67:188-205.

29. Strate LL. Lifestyle factors and the course of diverticular disease. Dig Dis 2012;30:35-45.

30. Appleby PN, Key TJ. The long-term health of vegetarians and vegans. Proc Nutr Soc 2015;1-7.

31. Carabotti M, Annibale B, Severi C, Lahner E. Role of Fibre in Symptomatic Uncomplicated Diverticular Disease: A Systematic Review. Nutrients 2017;9.

32. Unlu C, Daniels L, Vrouenraets BC, Boermeester MA. A systematic review of high-fibre dietary therapy in diverticular disease. Int J Colorectal Dis 2012;27:419-27.

33. Staudacher HM, Kurien M, Whelan K. Nutritional implications of dietary interventions for managing gastrointestinal disorders. Curr Opin Gastroenterol 2018;34:105-11.

Supplementary Table 2. Relative risks and 95% confidence intervals from nonlinear dose-response analysis

|  | Total dietary fibre |  | Cereal fibre |
| --- | --- | --- | --- |
| g/d | RR (95% CI) | g/d | RR (95% CI) |
|  |  | 1.7 | 1.00 |
| 7.5 | 1.00 | 5 | 1.19 (1.05-1.35) |
| 10 | 0.94 (0.93-0.96) | 10 | 0.99 (0.89-1.10) |
| 15 | 0.85 (0.83-0.88) | 15 | 0.90 (0.82-0.98) |
| 20 | 0.77 (0.74-0.79) | 20 | 0.84 (0.77-0.91) |
| 25 | 0.68 (0.65-0.72) | 25 | 0.80 (0.74-0.87) |
| 30 | 0.59 (0.55-0.64) | 30 | 0.78 (0.72-0.84) |
| 35 | 0.51 (0.45-0.58) |  |  |
| 40 | 0.42 (0.35-0.51) |  |  |
| p_nonlinearity_ | 0.35 | p_nonlinearity_ | 0.002 |

Supplementary Table 3. Newcastle-Ottawa study quality scores of each study included in the analysis

| Author | Representa-tiveness | Selection of non-exposed cohort | Exposure ascertainment | Demonstration that the outcome was not present at start | Adjustment for one confounding factor | Adjustment for a second confounding factor | Assessment of outcome | Long enough follow-up | Adequacy of follow-up | Total |
| --- | --- | --- | --- | --- | --- | --- | --- | --- | --- | --- |
| Aldoori, 1994 | 0 | 1 | 1 | 1 | 1 | 1 | 1 | 1 | 0 | 7 |
| Crowe, 2012 | 0 | 1 | 1 | 1 | 1 | 1 | 1 | 1 | 0 | 7 |
| Crowe, 2014 | 1 | 1 | 1 | 1 | 1 | 1 | 1 | 1 | 0 | 8 |
| Mahmood, 2018, SMC | 1 | 1 | 1 | 1 | 1 | 1 | 1 | 1 | 1 | 9 |
| Mahmood, 2018, COSM | 1 | 1 | 1 | 1 | 1 | 1 | 1 | 1 | 1 | 9 |

Supplementary Figure 1. Sensitivity analysis excluding one study at a time

------------------------------------------------------------------------------

Study omitted | e^coef. [95% Conf. Interval]

-------------------+----------------------------------------------------------

Mahmood, 2018, COSM| 0.74512422 0.71449971 0.77706128

Mahmood, 2018, SMC| 0.74073648 0.70994812 0.77285999

Crowe, 2014 | 0.76049829 0.69360650 0.83384109

Crowe, 2012 | 0.74465919 0.71307975 0.77763712

Aldoori, 1994 | 0.74116206 0.71014273 0.77353632

-------------------+----------------------------------------------------------

Combined | 0.74380320 0.71355226 0.77533663

------------------------------------------------------------------------------

Supplementary Figure 2. Funnel plot of fibre intake and diverticular disease
