# Supplementary material for: Dietary fibre intake and the risk of diverticular disease: a systematic review and meta-analysis of prospective studies
Source: Eur J Nutr. 2019 Apr 29;59(2):421–32. doi: 10.1007/s00394-019-01967-w (PMC7058673; doi:10.1007/s00394-019-01967-w)
Supplement: Supplementary file 2 — Supplementary material 2 (DOCX 19 kb) [file 394_2019_1967_MOESM2_ESM.docx]

Moose checklist_fibre and diverticular disease

| Reporting of background should include | Page |
| --- | --- |
| Problem definition | 3, 4 |
| Hypothesis statement | 3,4 |
| Description of study outcome(s) | 4 |
| Type of exposure or intervention used | 3,4 |
| Type of study designs used | 4 |
| Study population | 3-5 |
|  |  |
| Reporting of search strategy should include | |
| Qualifications of searchers (eg, librarians and investigators) | 5 |
| Search strategy, including time period included in the synthesis and keywords | 5 |
| Effort to include all available studies, including contact with authors | No contact with authors |
| Databases and registries searched | 5 |
| Search software used, name and version, including special features used (eg, explosion) | 5 |
| Use of hand searching (eg, reference lists of obtained articles) | 5 |
| List of citations located and those excluded, including justification | 5, Supplementary Table 1, Figure 1 |
| Method of addressing articles published in languages other than English | No relevant non-English articles identified |
| Method of handling abstracts and unpublished studies | 5 |
| Description of any contact with authors | No contact with authors |
|  |  |
| Reporting of methods should include | |
| Description of relevance or appropriateness of studies assembled for assessing the hypothesis to be tested | 5 |
| Rationale for the selection and coding of data (eg, sound clinical principles or convenience) | 5 |
| Documentation of how data were classified and coded (eg, multiple raters, blinding, and interrater reliability) | 5 |
| Assessment of confounding (eg, comparability of cases and controls in studies where appropriate) | 6 |
| Assessment of study quality, including blinding of quality assessors; stratification or regression on possible predictors of study results | 6 |
| Assessment of heterogeneity | 6 |
| Description of statistical methods (eg, complete description of fixed or random effects models, justification of whether the chosen models account for predictors of study results, dose-response models, or cumulative meta-analysis) in sufficient detail to be replicated | 5, 6 |
| Provision of appropriate tables and graphics | Table 1, Figure 1-3 |
|  |  |
| Reporting of results should include | |
| Graphic summarizing individual study estimates and overall estimate | 7, Figure 2, 3 |
| Table giving descriptive information for each study included | 7, Table 1 |
| Results of sensitivity testing (eg, subgroup analysis) | 8, Table 2 |
| Indication of statistical uncertainty of findings | 7,8 |
|  |  |
| Reporting of discussion should include | |
| Quantitative assessment of bias (eg, publication bias) | 9, 10 |
| Justification for exclusion (eg, exclusion of non–English-language citations) | No relevant non-English studies were identified |
| Assessment of quality of included studies | 11 |
|  |  |
| Reporting of conclusions should include | |
| Consideration of alternative explanations for observed results | 9-11 |
| Generalization of the conclusions (ie, appropriate for the data presented and within the domain of the literature review) | 9-11 |
| Guidelines for future research | 11 |
| Disclosure of funding source | 12 |
